# Recent advances in the therapeutic efficacy of hepatocyte growth factor gene‐modified mesenchymal stem cells in multiple disease settings

**DOI:** 10.1111/jcmm.17497

**Published:** 2022-08-03

**Authors:** Hong‐fang Meng, Jide Jin, Hua Wang, Li‐sheng Wang, Chu‐tse Wu

**Affiliations:** ^1^ School of Chemical Engineering and Technology, Tianjin University Tianjin China; ^2^ Beijing Key Laboratory for Radiobiology Beijing Institute of Radiation Medicine Beijing China

**Keywords:** clinical application, hepatocyte growth factor (HGF), *HGF* gene‐modified MSCs (HGF/MSCs), mesenchymal stem cells (MSCs), therapeutic efficacy

## Abstract

Mesenchymal stem cell (MSC) therapy is considered a new treatment for a wide range of diseases and injuries, but challenges remain, such as poor survival, homing and engraftment rates, thus limiting the therapeutic efficacy of the transplanted MSCs. Many strategies have been developed to enhance the therapeutic efficacy of MSCs, such as preconditioning, co‐transplantation with graft materials and gene modification. Hepatocyte growth factor (HGF) is secreted by MSCs, which plays an important role in MSC therapy. It has been reported that the modification of the *HGF* gene is beneficial to the therapeutic efficacy of MSCs, including diseases of the heart, lung, liver, urinary system, bone and skin, lower limb ischaemia and immune‐related diseases. This review focused on studies involving HGF/MSCs both in vitro and in vivo. The characteristics of HGF/MSCs were summarized, and the mechanisms of their improved therapeutic efficacy were analysed. Furthermore, some insights are provided for HGF/MSCs' clinical application based on our understanding of the *HGF* gene and MSC therapy.

## 1 INTRODUCTION

Mesenchymal stem cells (MSCs) are multipotent postnatal stromal cells that can be isolated from various adult tissues, such as bone marrow (BM),^1^ umbilical cord (UC),^2^ umbilical cord blood (UCB),^3^ adipose tissue (AD)^4^ and dental pulp.^5^ MSCs are plastic adherent and can be expanded in vitro. They possess several features, including self‐renewability, multipotency, immune evasion or privilege, homing and immune regulation. MSC therapy has been adopted in various conditions, such as ageing frailty,^6^ inflammatory diseases,^7^ lung diseases,^8^ liver diseases,^9^ renal diseases^10^ and neurodegenerative diseases.^11^ There were approximately 1300 studies of MSC therapy registered on ClinicalTrials.gov by the end of 2021. MSCs have been generally proven to be safe and effective. Although significant progress has been made in MSC therapy, several hurdles limit their therapeutic efficacy, such as poor survival, homing and low engraftment rates. Thus far, many strategies have been developed to enhance the therapeutic efficacy of MSCs, such as preconditioning, co‐transplantation with graft materials and gene modification. Gene modification could enhance MSCs' characteristics and improve the production of desirable, beneficial gene products, ultimately optimizing the therapeutic efficacy of MSCs.^12^, ^13^, ^14^

Hepatocyte growth factor (HGF) is a pleiotropic factor primarily secreted by mesenchymal cells^15^ that was first identified and cloned in the 1980s.^16^, ^17^ HGF has mitogenic, motogenic, anti‐apoptotic, morphogenic and immune regulation activities,^18^, ^19^ which can prevent fibrosis, apoptosis and inflammation, and promote angiogenesis in multiple conditions.^20^ It has been proven that MSCs' therapeutic efficacy completely or partially depends on the secretion of HGF.^21^, ^22^, ^23^, ^24^, ^25^ In 2003, our group first reported research about *HGF* gene‐modified MSCs, in which the MSCs' therapeutic efficacy on myocardial ischaemia was improved by *HGF* gene modification.^26^ Since then, dozens of studies about *HGF* gene‐modified MSCs have been conducted. This review summarizes the characteristics of *HGF* gene‐modified MSCs (HGF/MSCs). In addition, the mechanisms of their enhanced therapeutic efficacy were analysed, thus giving some insights into their clinical application.

## 2 CHARACTERISTICS OF HGF/MSC IN VITRO

Up to now, seven kinds of *HGF* gene modification vectors have been used in preclinical studies, namely Ad‐HGF, adeno‐associated virus vector carrying *HGF* gene (AAV‐HGF), lentivirus vector carrying *HGF* gene (Lenti‐HGF), retrovirus vector carrying *HGF* (Retro‐HGF), HGF plasmid, transcription activator‐like effector nucleases (TALEN) system and gene‐delivery nano‐system (Table S1). Ad‐HGF, AAV‐HGF, plasmid and gene‐delivery nano‐systems use non‐integrating vectors, and Lenti‐HGF, Retro‐HGF and TALEN systems use integrating vectors. The definition of cell characteristics is vital for stem cell‐based therapeutic products. In 2006, the International Society for Cellular Therapy defined the minimal criteria characteristics of MSCs.^27^ For HGF/MSC application, it is important to clarify whether the HGF gene modification has changed these characteristics. According to the preclinical studies, the MSCs' phenotype was not changed by *HGF* gene modification. In the HGF/MSCs, the stem cell markers CD105, CD73 and CD90 are still positive, but the haematopoietic markers CD34, CD45, CD11b, endothelial marker CD31 and major HLA II are negative. The expression percentages of these markers in HGF/MSCs are the same as in the unmodified MSCs.^28^, ^29^, ^30^, ^31^, ^32^, ^33^, ^34^, ^35^, ^36^, ^37^ Also, HGF/MSCs were as multipotent as MSCs. They were capable of adipogenesis, osteogenesis and chondrogenesis under appropriate induction.^28^, ^30^, ^31^, ^34^, ^38^, ^39^ Some studies indicated that *HGF* gene modification by Ad‐HGF vector might enhance MSCs' osteogenic and neurogenic differentiation abilities. The expression of osteogenic differentiation‐related genes encoding alkaline phosphatase (ALP), runt‐related transcription factor 2 (*Runx2*) and osteocalcin (OC) in dental pulp stem cells (DPSCs),^40^ and the expression of dopaminergic neuron‐related genes tyrosine hydroxylase (*TH*), dopamine transporter (DAT) and dopamine (DA) in UC‐MSCs^41^ were all upregulated by Ad‐HGF modification. This enhancement might depend on the multiplicity of infection (MOI) dosage. It was confirmed that the formation of mineralized extracellular matrix (ECM) in BM‐MSCs infected with Ad‐HGF at MOI = 10 or 50 was not significantly different from BM‐MSCs infected with adenovirus vector lack of exogenous genes (Ad‐Null). However, a significantly enhanced osteogenic differentiation was observed in BM‐MSCs infected with Ad‐HGF, which was higher than in those infected with Ad‐Null when the MOI was elevated to 250.^42^

## 3 HGF OVEREXPRESSION BY HGF/MSCS

Although HGF/MSCs' characteristics did not show obvious differences by different modification vectors, the HGF overexpression varied (Table 1). Ad‐HGF's infection acting time was the shortest; the HGF expression peak appeared at day 2 post‐infection, and the overexpressing time was maintained for about 14 days.^26^, ^42^, ^43^, ^44^, ^45^ AAV‐HGF's infection acting time was the longest; the HGF expression peak appeared at day 11 post‐infection, while the overexpressing‐maintained time was the longest, too, about 31 days.^46^ However, when the HGF/MSCs fabricated by different MSCs and HGF vectors were transplanted in vivo, they showed similar overexpressing‐maintained time, about 3–4 weeks.^29^, ^47^, ^48^, ^49^, ^50^, ^51^, ^52^ The advanced therapeutic effect was maintained for about the same period,^29^, ^33^, ^46^, ^53^, ^54^, ^55^, ^56^ indicating that the in vivo survival of HGF/MSCs was barely affected by the MSC source and HGF modification method.

**TABLE 1:** HGF overexpression by HGF/MSCs

Vector	MSC source	HGF in vitro expression			HGF in vivo expression		Advanced therapeutic effect maintains
		Peak	Half peak	Maintain	Peak	Maintain	
Adenovirus	BM‐MSC	At day 2^26^, ^42^, ^43^, ^44^, ^45^, ^76^	At day 7–8^26^, ^42^, ^43^, ^44^, ^45^, ^76^	At least 14 days^26^, ^42^, ^44^, ^45^	At day 1^44^	At least 28 days^29^, ^47^, ^48^, ^49^	At least 28 days^29^, ^53^
Adenovirus	UC‐MSCs	At day 2–4^94^	–	At least 3 days^94^	–	At least 28 days^50^	At least 28 days^54^ at least 21 days^55^
AAV	AD‐MSC	At day 11^46^	At day 14–15^46^	At least 31 days^46^	–	–	At least 21 days^46^
Lentivirus	BM‐MSC	–	–	–	–	At least 21 days^51^	At least 28 days^33^
pMEX plasmid	UCB‐MSC	–	–	At least 2 days^52^, ^105^, ^106^	–	At least 28 days^52^	At least 28 days^56^
pIRES2 plasmid	Endothelial progenitor cells	At day 3^67^	At day 9^67^	At least 9 days^67^	–	–	–
Spermine‐pullulan plasmid	BM‐MSC	At day 3^77^	At day 4–5^77^	At least 7 days^77^	–	–	At least 28 days^77^

Abbreviations: HGF, hepatocyte growth factor; MSC, mesenchymal stem cell; HGF/MSC, HGF gene‐modified MSC; AAV, adeno‐associated virus vector; BM, bone marrow; UC, umbilical cord; UCB, umbilical cord blood; AD, adipose tissue; DPSC, dental pulp stem cell; VS., versus; =, equal; ↑, improved; –, not mentioned.

## 4 THERAPEUTIC EFFICACY OF HGF/MSCS IN PRECLINICAL STUDIES

**FIGURE 1:**
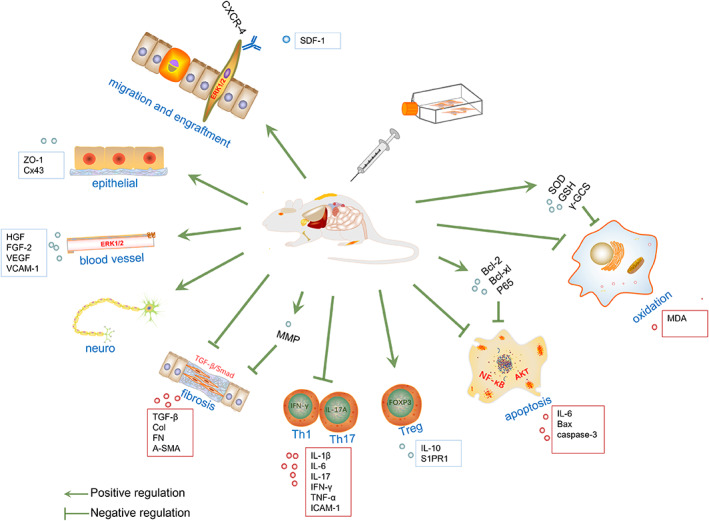
Main mechanisms of HGF/MSC therapy. HGF/MSCs were adopted in treatment for a variety of diseases, including ischaemic, heart, lung, liver, urinary system, bone and immune‐related diseases. (1) HGF/MSC promote cell migration and engraftment, in which SDF‐1α/CXCR‐4 axis and ERK1/2 signalling pathway were involved. (2) HGF/MSCs promote cell–cell connection restoration and soft tissue re‐epithelialization. (3) HGF/MSCs promote angiogenesis and neurogenesis. (4) HGF/MSCs promote anti‐fibrosis effect. (5) HGF/MSCs promote anti‐inflammatory effect. They can deactivate Th1 and Th17 cells and activate Treg cells. (6) HGF/MSCs promote anti‐apoptosis effect. (7) HGF/MSCs promote anti‐oxidation effect. Bcl, B‐cell lymphoma; COL, collagen; Cx43, connexin 43; CXCR4, chemokine (C‐X‐C motif) receptor 4; ERK1/2, extracellular regulated protein kinases 1/2; FGF, fibroblast growth factor; FN, fibronectin; GSH, antioxidant glutathione; ICAM, intercellular adhesion molecule; IFN‐γ, interferon gamma; IL, interleukin; MDA, antioxidant metabolite malondialdehyde; MMP, matrix metalloproteinase; S1PR1, sphingosine 1‐phosphate receptors 1; SDF‐1, stromal cell‐derived factor‐1; Smad, small mothers against decapentaplegic; SOD, superoxide dismutase; TGF‐β, transforming growth factor‐beta; Th1, T helper 1 cell; Th17, interleukin 17 (IL‐17)‐secreting helper T; TNF‐α, tumour necrosis factor alpha; Treg, regulatory T cell; VCAM, vascular cell adhesion protein; VEGF, vascular endothelial growth factor; ZO‐1, zonula occludens‐1; α‐SMA, alpha‐smooth muscle Actin; γ‐GCS, gama glutamylcysteine synthetase.

HGF/MSCs showed a synergic therapeutic effect of MSC and HGF (Table 2). Both MSC therapy and HGF protein/gene therapy were beneficial to angiogenesis, organ structure recovery, organ function recovery and anti‐fibrosis. In contrast, HGF/MSC therapy was more effective than either alone.^26^, ^44^, ^47^, ^50^, ^57^, ^58^, ^59^ This review summarized 49 preclinical studies that applied HGF/MSC therapy in various disease settings, such as myocardial infarction, hindlimb ischaemia, liver/kidney fibrosis, pulmonary arterial hypertension, acute lung/kidney injury, osteoporosis and immune‐related diseases. They all showed that HGF/MSCs had advanced therapeutic efficacy compared with MSCs or HGF protein/gene therapy (except for one study,^60^ which is mentioned in the ‘Discussion and perspectives’ section). The details about the preclinical studies of HGF/MSC therapy are summarized in Table S1. The main mechanisms of HGF/MSC therapy are summarized in Figure 1, and they are further demonstrated below.

**TABLE 2:** Therapeutic efficacy of HGF/MSC compared with MSC and HGF protein/gene

Disease	Cells	HGF	Therapeutic effect		
			HGF/MSCs vs. control	HGF/MSCs vs. MSCs	HGF/MSCs vs. HGF
Myocardial ischaemia^26^	5 × 10^6^	1 × 10^8^ pfu Ad‐HGF	↓ Collagen content↑ capillaries count↓ infarct size↑ heart functions	\ Collagen content↑ capillaries count\ infarct size\ heart functions	↓ Collagen content↑ capillaries count\ infarct size\ heart functions
Myocardial infarction^57^	2 × 10^6^	2.1 × 10^7^ cfu retro‐HGF	↓ Infarct size ↑ anterior wall thickness ↑ vascular density ↑ heart function	↓ Infarct size = anterior wall thickness ↑ vascular density ↑ heart function	↓ Infarct size ↑ anterior wall thickness↑ vascular density↑ heart function
Hindlimb ischaemia^59^	2 × 10^7^	2 μg protein	↑ Blood flow↑ capillaries count↑ micro vessels count↑ endothelial thickness	↑ Blood flow↑ capillaries count↑ micro vessels count↑ endothelial thickness	↑ Blood flow\ capillaries count\ micro vessels count\ endothelial thickness
Hindlimb ischaemia^58^	0.3/1/10 × 10^5^	1/10/100 × 10^6^ pfu Ad‐HGF	↑ Blood flow ↑ capillaries and arterioles count	↑ Blood flow ↑ capillaries and arterioles count	↑ Blood flow ↑ capillaries and arterioles count
Liver transplant^44^	5 × 10^6^	1 × 10^9^ pfu Ad‐HGF	↓ Mortality rate↑ liver weight↑ liver function↑ hepatocytes proliferation↓ hepatocytes apoptosis	↓ Mortality rate↑ liver weight↑ liver function↑ hepatocytes proliferation↓ hepatocytes apoptosis	↓ Mortality rate = liver weight↑ liver function↑ hepatocytes proliferation↓ hepatocytes apoptosis
Liver transplant^47^	5 × 10^6^	1 × 10^9^ pfu Ad‐HGF	↓ Fibrosis↑ hepatocytes proliferation↓ hepatocytes apoptosis↓ hepatic stellate cells activities↑ liver function	↓ Fibrosis↑ hepatocytes proliferation↓ hepatocytes apoptosis↑ hepatic stellate cells activities↑ liver function	↓ Fibrosis↑ hepatocytes proliferation↓ hepatocytes apoptosis↓ hepatic stellate cells activities↑ liver function
Sinonasal wound^50^	6 × 10^6^	1 × 10^9^ pfu Ad‐HGF	↑ Wound healing↓ collagen deposition↑ cilia recovery	↑ Wound healing↓ collagen deposition↑ cilia recovery	↑ Wound healing↓ collagen deposition↑ cilia recovery

Abbreviations: HGF, hepatocyte growth factor; Ad‐HGF, adenovirus vector carrying HGF gene; retro‐HGF, retrovirus vector carrying HGF; VS., versus; =, equal; ↑, improved; ↓, reduced; \, not mentioned.

## 5 PROMOTING ENGRAFTMENT AND TISSUE REPAIRMENT

The proliferation and migration activities of the transplanted MSCs are related to their in vivo engraftment efficiency. HGF is a mitogen factor involved in organ development and regeneration.^20^ In preclinical studies, *HGF* gene modification enhanced the therapeutic effect of MSCs on the organ structure recovery through enhancing soft tissue re‐epithelialization, restoring cell–cell connection and promoting hard tissue regeneration. Epithelial cells are widely distributed between the external and internal surfaces of the host, thus playing an important role in organ physiological homeostasis.^61^ HGF/MSCs promoted the activities and reduced the apoptosis of epithelial cells in the diseased organs, including the injured intestine,^54^ transplanted trachea,^55^ involute thymi^37^ and burned skin.^62^ Cells are connected by tight junctions and gap junctions, while the tight junctions are only found among epithelial cells.^63^ Zonula occludens‐1 (ZO‐1) is a major component of tight junctions, and connexin 43 (Cx43) is a kind of gap junction protein.^64^ The expression of ZO‐1^54^ and Cx43^65^ was upregulated by HGF/MSCs more efficiently than only MSCs. Other than enhancing re‐epithelialization and restoring the cell–cell connection, the bone regeneration was promoted by HGF/MSCs more than only MSCs in the diseased microenvironments.^38^, ^40^, ^48^

## 6 PROMOTING ANGIOGENESIS

All tissues should be nurtured by the extensive networks formed by blood vessels. The progression of various diseases correlates with tissue destruction, such as necrosis and ischaemic and inflammatory diseases.^66^ It was reported that HGF/MSCs promoted vascular endothelial cell proliferation and blood vessel regeneration more efficiently than MSCs and HGF protein.^46^, ^48^, ^51^, ^58^, ^59^, ^67^, ^68^ The endothelial marker CD31 was expressed by the transplanted HGF/BM‐MSCs in the model of rats with hindlimb ischaemia,^51^ suggesting that the transplanted HGF/MSC might differentiate into endothelial cells in the host. Moreover, HGF/MSC also can upregulate the expression of proangiogenic cytokines. In mice with hindlimb ischaemia, FGF‐2 expression in the limb can be induced by HGF/BM‐MSCs and higher than BM‐MSCs.^58^ HGF/MSCs might promote angiogenesis through the ERK1/2 signalling pathway. HGF/MSC promoted the expression of phosphorylated ERK1/2 both in vitro^67^ and in vivo,^48^ and the treatment with an ERK1/2 inhibitor decreased the capillary‐like structures.^67^ Moreover, the expression of sphingosine 1‐phosphate receptors 1 (S1PR1), one of the downstream proteins of the ERK1/2 signalling pathway, was upregulated by HGF/BM‐MSCs treatment in the injured lung.^29^

## 7 PROMOTING NEUROGENESIS

Both MSC and HGF are beneficial to neurogenesis. In addition, *HGF* gene modification could enhance the expression of the dopaminergic neuron‐related genes *TH, DAT* and *DA* in UC‐MSCs.^41^ Three studies about HGF/MSCs addressed their therapeutic efficacy on the neural system. These studies showed that HGF/MSCs promoted the re‐innervation in infarcted heart^69^ and ischaemic limb^46^ more efficiently than MSCs. Also, significantly more myelinated fibres were present in in intracerebral haemorrhage rats transplanted with HGF/UC‐MSCs than with UC‐MSCs.^39^ Therefore, the HGF gene modification enhanced the MSC potential of neurogenesis in vivo.

## 8 PROMOTING ANTI‐FIBROSIS EFFECT

Fibrosis is defined by the accumulation of excess ECM components, which can affect any organ and is responsible for up to 45% of all deaths in the industrialized world.^70^ It has been reported that HGF/MSCs could reduce fibrosis efficiently in various diseased organs, including the heart,^68^, ^71^ lung,^29^, ^55^, ^72^, ^73^, ^74^ liver,^47^, ^52^, ^56^, ^75^, ^76^, ^77^, ^78^ kidney,^45^, ^49^ bladder,^79^ bone^48^ and skin.^62^ Collagen (COL) is the most important component of ECM, hydroxyproline is the most important component of COL, and fibronectin (FN) is the most important non‐collagenous component of ECM. It has been shown that HGF/MSCs could reduce the expression of COL, hydroxyproline and FN more efficiently than MSCs.^49^, ^50^, ^62^, ^72^ The matrix metalloproteinase (MMP) family can degrade a wide spectrum of extracellular matrix proteins, such as fibronectin and laminins. It has been reported that HGF/BM‐MSCs transplantation could upregulate the expression of MMP‐14 and downregulate the expression of the tissue inhibitors of MMP‐1 (TIMP‐1) in the liver fibrosis, whereas BM‐MSCs cannot.^76^ Therefore, HGF/MSCs could inhibit fibrosis by reducing ECM accumulation and promoting ECM degradation. Alpha‐smooth muscle actin (α‐SMA) indicates activated hepatic stellate cells (HSCs) and fibroblasts, which are the major source of ECM. It was demonstrated that the expression of α‐SMA was reduced by HGF/MSC transplant more than when only MSCs were transplanted.^45^, ^47^, ^49^, ^75^, ^78^ HGF/MSC administration could induce more HSC apoptosis than MSCs and Ad‐HGF in liver fibrosis.^47^ Hence, HGF/MSCs could suppress the activities of ECM‐producing cells in fibrosis. HGF/MSCs downregulated the expression of transforming growth factor‐beta (TGF‐β) more efficiently than MSCs^29^, ^55^, ^74^, ^76^, ^78^ and Ad‐HGF.^47^, ^50^ TGF‐β is the key activator of fibroblasts and the central cellular effector of fibrotic responses, which exerts its biological effects by activating downstream mediators, including small mothers against decapentaplegic (DPP) Smad2 and Smad3, is negatively regulated by Smad7 expression.^80^ In the rats with liver fibrosis, the HGF/UC‐MSC transplant downregulated the expression of TGF‐β1, Smad2 and Smad3 more efficiently than UC‐MSCs,^78^ suggesting that the advantageous therapeutic efficacy of HGF/MSCs might depend on the TGF‐β/Smad signalling pathway.

## 9 PROMOTING ANTI‐INFLAMMATORY EFFECT

Both MSCs and HGF possess anti‐inflammatory properties. Inflammation is a complex set of interactions in response to traumatic, infectious, post‐ischaemic, toxic or autoimmune injuries, which can lead to persistent tissue damage by leukocytes, lymphocytes or collagen.^81^ It was reported that HGF/MSCs were beneficial to the survival of the graft in the host.^43^, ^44^, ^47^, ^55^ The degree of inflammatory infiltration and the expression of infiltration indicators, such as intercellular adhesion molecule (ICAM)‐1 and myeloperoxidase (MPO), were suppressed by the HGF/MSCs more than by the MSCs.^28^, ^29^, ^47^ Moreover, the pro‐inflammatory factors IL‐1β, interferon‐gamma (IFN‐γ), TNF‐α and IL‐17A were more downregulated by HGF/MSCs transplantation, whereas the anti‐inflammatory factors IL‐4 and IL‐10 were more upregulated.^28^, ^29^, ^30^, ^31^, ^32^, ^54^, ^55^ IFN‐γ and TNF‐α are two of the cytokines mainly expressed by Th1 cells, IL‐17A is one of the cytokines mainly expressed by Th17 cells, IL‐4 is one of the cytokines mainly expressed by (T helper 2) Th2 cells, and IL‐10 is one of the cytokines mainly expressed by Treg cells. When co‐cultured with lymphocytes in vitro, HGF gene modification did not change the suppression effect of MSCs on the stimulated lymphocyte proliferation^32^, ^43^ and did not change the activation effect of MSCs on the monocytes,^43^ but enhanced the suppression effect of MSCs on the activities of Th1 and Th17 cells, and enhanced the promotion effect of MSCs on the Treg cell activities.^32^ HGF/MSCs decreased the ratio of Th1 to Th2 cells in the spleen more efficiently than the MSCs,^55^ and downregulated the expression of transcription factor Th1, T‐box transcription factor 21 (T‐bet) and Th17 transcription factor retinoic acid‐related orphan receptor‐γt (RORγt), but upregulated Treg transcription factor Foxp3 more efficiently.^32^ Above all, HGF/MSCs might reduce the inflammatory responses through regulating the polarization and activities of CD4^+^ T cells.

## 10 PROMOTING ANTI‐APOPTOSIS EFFECT

*HGF* gene modification not only could enhance the anti‐apoptosis potential of MSCs in the microenvironments of hypoxia or inflammation in vitro, but it also could enhance their suppression effect on the apoptosis of parenchymal cells in vivo, such as cardiomyocytes,^34^, ^69^, ^82^ lung epithelial cells,^28^, ^29^ hepatocytes,^44^, ^47^, ^75^ renal cells^30^ and intestinal epithelial cells.^54^ It was reported that the expression of caspase‐3 was suppressed by HGF/MSCs more than when only MSCs were used.^30^, ^35^ Caspases are proteolytic enzymes known largely for controlling cell death and inflammation. The apoptotic caspases are subdivided into the initiators and the effectors. Initiator caspase activation during apoptosis is mediated mainly by the mitochondrial (intrinsic) and the death receptor (extrinsic) pathways. The intrinsic pathway is regulated by pro‐apoptotic B‐cell lymphoma (BCL)‐2 homology domain 3 (BH3) of the members (Bim, Bid, Puma, Noxa, Hrk, Bmf and Bad), pro‐apoptotic effector molecules (Bax and Bak) and anti‐apoptotic Bcl‐2 family proteins (Bcl‐2, Bcl‐xL, Mcl1, A1 and Bcl‐B). Once initiator caspases are activated through the extrinsic or intrinsic apoptosis pathways, they mediate the activation of effector caspases, leading to cell structure destruction and apoptosis, and caspase‐3 is one of the effector caspases.^83^ It was reported that HGF/MSCs upregulated the expression of Bcl‐2^31^, ^35^, ^69^, ^82^, ^84^ and Bcl‐xL,^35^ and downregulated the expression of BCL‐2‐associated X (Bax)^31^, ^69^ more efficiently than MSCs, indicating that HGF/MSCs could suppress the apoptosis through deactivation of the mitochondrial pathway. In addition, the expression of AKT^35^, ^82^ and p65^31^ was increased by HGF/MSCs treatment more than the MSCs. Akt can rescue the cells from apoptosis by the activation of anti‐apoptotic factors, such as glycogen synthase kinase‐3 (GSK3), Bcl‐2, and inactivation of pro‐apoptotic factors, such as BCL2‐associated agonist of cell death (Bad), caspase‐9, and forkhead (FH) transcription factors.^85^ The heterodimer of p65 and p50 is the most abundant and canonical form of NF‐κB. NF‐κB has anti‐apoptotic functions by downregulating the inflammation response.^86^ To sum up, HGF/MSCs could suppress the apoptosis of parenchymal cells by deactivating the mitochondrial (intrinsic) pathway, in which the AKT and NF‐κB signalling pathways might be involved.

## 11 PROMOTING ANTI‐OXIDATION EFFECT

Oxidative stress is implicated in various chronic/degenerative diseases, resulting in macromolecular damage.^87^ There are two kinds of oxidant compounds, namely reactive oxygen species (ROS) and reactive nitrogen species (RNS), which introduce various oxidative insults to lipids, proteins and nucleic acids, with consequences ranging from subtle modulation of cell signal transduction processes to apparent biomolecular damage and cell death.^88^ The antioxidant system is composed of nonenzymatic antioxidants and enzymatic antioxidants. The nonenzymatic antioxidants are low molecular weight compounds, including glutathione (GSH), vitamin C and β‐carotene. The enzymatic antioxidants can be divided into two groups: the antioxidant response element‐driven enzymes and primarily or constitutively acting antioxidant enzymes, such as superoxide dismutase (SOD), catalase and GSH peroxidase.^89^ It was reported that HGF/MSCs could upregulate the expression of SOD and downregulate the expression of malondialdehyde (MDA), GSH and γ‐glutamyl cysteine synthetase (γ‐GCS) more efficiently than the MSCs when transplanted in vivo.^28^, ^31^ MDA is an indicator for lipid peroxidation, and γ‐GCS is a rate‐limiting enzyme of GSH synthesis within the cell. Therefore, HGF/MSCs could reduce oxidative stress by decreasing lipid peroxidation, probably through their ability to promote the activity of SOD and the synthesis of GSH.

## 12 THERAPEUTIC EFFICACY OF HGF/MSC APPLICATION IN A CLINICAL STUDY

Silicosis is an irreversible disease characterized by lung fibrosis. A clinical study on HGF/MSCs therapy for silicosis concluded that the administration of HGF/BM‐MSCs was safe and effective in some patients with silicosis. Briefly, HGF/BM‐MSCs were prepared by transfecting autologous BM‐MSCs with plasmid HGF. Then, HGF/BM‐MSCs were administered intravenously to four patients with pulmonary silicosis at a dose of 2 × 10^6^ cells/kg weekly for three consecutive weeks. Two patients had dexamethasone‐relievable fever after the administration, but no other abnormal symptoms were observed after the treatment for 6 months. The lung function indicators, such as forced vital capacity (FVC), the forced expiratory volume averages at the first second (FEV1) and the arterial blood oxyhemoglobin saturation (SpO_2_), were improved; the ratios of peripheral blood CD4^+^/CD8^+^ cell concentrations were increased; the serum IgG levels were decreased to the normal range; and the average ceruloplasmin level was slightly decreased, indicating an improvement of lung function and a reduction of inflammation. Furthermore, the absorption of the nodular lesion was observed after treatment for 12 months in 2 patients, suggesting structural healing from the silicotic fibrosis.^90^

## 13 DISCUSSION AND PERSPECTIVES

Gene‐modified stem cells could be applied to the next generation of cell‐based therapies. How to screen for the gene and cell source, the modification process, and the indications for the modified cell suitable for the therapies are three basic questions that need to be answered in gene‐modified stem cell therapy. In Table 2, HGF/MSCs prepared by different MSCs and HGF vectors showed a similar overexpression lasting time in vivo. Therefore, in vitro infection efficiency and in vivo safety are the key factors for selecting a vector, cell source and modification processes. For now, the adenovirus is the most efficient vector for HGF gene modification. Ad‐HGF modification did not change the cell genome, and the characteristics of Ad‐HGF‐modified cells are similar and fit the minimal criteria definition of MSCs. Therefore, MSCs are safe for clinical application at present, and in this review, no safety issues about HGF/MSCs occurred. However, HGF is a tumour growth promotion factor. Therefore, the issue of HGF/MSCs safety still needs to be further explored.

This review summarizes 49 preclinical studies on HGF/MSC therapy. These studies demonstrated that HGF/MSCs showed a more conspicuous therapeutic efficacy than MSCs or HGF protein/gene therapy. Except for one report, HGF/DPSCs and DPSCs were confirmed to possess an equal therapeutic effect on rheumatoid arthritis in mice within the first 41 days. However, HGF/DPSCs disappeared on day 41 after the administration, while the therapeutic effect of DPSCs was maintained.^60^ The reason might be that HGF played a role in joint angiogenesis and cartilage/bone destruction.^91^ Therefore, HGF/MSCs are only suitable for the scenarios in which both MSCs and HGF are beneficial. MSCs can attenuate neuroinflammation, reduce neural degeneration, promote neural regeneration, nourish and protect neurons and preserve the blood–brain barrier.^92^ HGF is involved in the development of nervous system from prenatal to adult life, and HGF also attenuates neuroinflammation, reduces neurodegeneration, promotes neuro‐regeneration, and nourishes and protect neurons.^93^ Hence, HGF/MSCs are beneficial to the recovery from nervous system diseases. However, few studies have adopted HGF/MSCs to treat neural diseases. It was shown that the culture supernatant of HGF/UC‐MSCs could promote neural regeneration, reduce intracellular free calcium levels and promote the intracellular levels of bound calcium in a Parkinson's disease cell model.^94^ Also, the beneficial effect of HGF/UC‐MSCs on myelination was confirmed in an intracerebral haemorrhage rat model.^39^ Hence, the therapeutic efficacy of HGF/MSCs on neural diseases could be further explored.

The prominent therapeutic effect of HGF/MSCs comes from both HGF and MSC. HGF has angiogenesis, anti‐fibrosis and anti‐inflammation properties; the overexpression of HGF in vivo is beneficial to many conditions. HGF gene modification promotes the in vivo survival rate of MSC, hence improving the MSCs' therapeutic effect. Furthermore, HGF/MSCs' therapeutic efficacy could be enhanced by prolonging the time for HGF overexpression by using HGF/MSCs cell sheet technology^46^ or HGF inducible microgel preparation.^59^ Theoretically, multiple doses could also enhance HGF/MSCs' therapeutic efficacy. Only two preclinical studies adopted multiple dosages of HGF/MSCs^52^, ^77^; however, the authors did not compare the therapeutic efficacy with single doses. Therefore, the optimal dosage of HGF/MSCs needs to be explored further.

The HGF/MSC therapy mechanism is essential for the indication choice and its clinical application, thus being worth further and deeper exploration. Except for the mechanisms of HGF/MSC therapy mentioned in the preclinical studies, there probably were some unexplored mechanisms for the advanced therapeutic efficacy of HGF/MSCs; for example, (1) the death receptor (extrinsic) pathway might be involved in the anti‐apoptosis effect of HGF/MSCs. Death receptor‐mediated apoptosis is initiated following ligand‐binding and activation of the death domain‐containing tumour necrosis receptor superfamily, such as CD95 (Fas).^83^ At the same time, MSCs might inhibit the host cell apoptosis through the Fas ligand (FasL)/Fas‐mediated death pathway.^95^ In addition, HGF can promote cell survival by inhibiting Fas activation‐mediated apoptosis.^96^ Hence, HGF/MSCs suppressed the apoptosis of parenchymal cells through the mitochondrial (intrinsic) pathway and might also through the death receptor (extrinsic) pathway. (2) HGF gene modification might affect mitochondrial activities. Mitochondria are the energy‐producing dynamic double‐membraned organelles essential for cellular and organismal survival. Both the advanced anti‐apoptosis and anti‐oxidation effects of HGF/MSCs correlate with mitochondrial activities. Mitochondria can be transferred between transplanted MSCs and damaged host cells to regulate their biological functions, such as cellular metabolism, survival, proliferation and differentiation.^97^ Also, MSC‐derived extracellular vesicles could attenuate the mitochondrial damage of the host cell.^98^ Whether the gene modification could affect the mitochondrial quality of MSCs, the host cells through mitochondrial transfer, and regulate the mitochondria quality of the host cells or not still need to be explored. (3) In this review, we demonstrated that HGF/MSC could reduce oxidation, apoptosis and inflammation. Oxidative stress could induce senescence^99^; senescence evolved alongside apoptosis^100^ and contributed to inflammation.^101^ Therefore, HGF/MSC might be anti‐senescence. It should be explored whether HGF modification could reduce MSC senescence and whether HGF/MSC could decelerate host senescence. Furthermore, there were other cytokines, such as fibroblast growth factor 21 (FGF21),^102^ stem cell factor (SCF)^103^ and Erb‐B2 receptor tyrosine kinase 4 (ERBB4),^104^ that could reduce senescence and apoptosis. Exploring the common and unique properties of HGF compared with these cytokines might be beneficial to the application of HGF/MSC. (4) The in vivo microenvironment affects HGF/MSCs' function. There were some controversial results of HGF/MSC therapy, such as HGF/MSC therapy decreased the expression of Col I in the trabeculae, but increased its expression in medullary cavities^48^; decreased the expression of α‐SMA in fibrosis (Table 2), but increased its expression in the cavernous tissue.^35^ The reason might be that the behaviour and activities of the transplanted HGF/MSCs could be affected by the surrounding physical (e.g. stiffness, elasticity, viscosity, hypoxia, fluid shear stress, hydrostatic pressure, bioelectricity and microgravity), chemical (e.g. ECM, chemokines and enzymes) and cellular (e.g. parenchymal cells, nonparenchymal cells and immune cells) microenvironments. To determine the changes in cell behaviours and activities between MSCs and gene‐modified MSCs under the same microenvironment would provide new strategies for cell therapy.

## AUTHOR CONTRIBUTIONS

**Hongfang Meng:** Conceptualization (lead); writing – original draft (lead). **Jide Jin:** Writing – review and editing (equal). **Hua Wang:** Writing – review and editing (equal). **Li‐sheng Wang:** Writing – review and editing (equal). **Chu‐tse Wu:** Project administration (lead); writing – review and editing (lead).

## Funding information

The authors declare that no funds, grants or other support were received during the preparation of this manuscript.

## CONFLICT OF INTEREST

The authors confirm that there are no conflicts of interest.

## Supporting information


Table S1
