# Supplementary material for: Recent advances in the therapeutic efficacy of hepatocyte growth factor gene‐modified mesenchymal stem cells in multiple disease settings
Source: J Cell Mol Med. 2022 Aug 3;26(18):4745–55. doi: 10.1111/jcmm.17497 (PMC9465188; doi:10.1111/jcmm.17497)
Supplement: Supplementary file 1 — Table S1 [file JCMM-26-4745-s001.docx]

# Table S1 The HGF/MSC therapy mechanisms in pre-clinical studies

| **Organ** | **Indication** | **Vector** | **MSC source** | **Animal** | **Treatment** | **Mechanism** |
| --- | --- | --- | --- | --- | --- | --- |
| heart | myocardial infarction ^82,107^ | adenovirus | rat BM-MSC | rat | intramyocardial injection,  5×10^6^ cells,  once | ↑ heart structure recovery  ↓ fibrosis (↓scar area, ↑MMP-9) ↑ angiogenesis (↑vessel densities, ↑VCAM-1) ↑ cardiomyocytes proliferation  ↓ cardiomyocytes apoptosis (↑Bcl-2) ↑ p-Akt expression |
|  | myocardial infarction ^71^ | adenovirus | swine BM-MSC | swine | retrograde coronary vein infusion,  1×10^8^ cells,  once | ↑ engraftment efficiency  ↑ heart structure recovery  ↓ fibrosis (↓scar area)  ↑ angiogenesis (↑vessel densities, ↑HGF, ↑VEGF) |
|  | myocardial infarction ^69^ | adenovirus | human MSC | swine | intramyocardial injection,  5×10^7^ cells,  once | ↑ angiogenesis (↑vessel densities)  ↑ neurogenesis  ↓ cardiomyocytes apoptosis (↑Bcl-2)  ↑ p-Akt expression |
|  | myocardial infarction ^34^ | lentivirus | human UC-MSC | mouse | intramyocardial injection,  3×10^5^ cells,  once | ↑ engraftment efficiency  ↓ fibrosis (↓scar area)  ↑ cardiomyocytes proliferation  ↓ cardiomyocytes apoptosis |
|  | myocardial infarction ^33^ | lentivirus | human BM-MSC | rat | loaded into  hdECM patches and implanted in hearts,  1×10^6^ cells,  once | ↓ fibrosis  ↑ cardiomyocytes activities |
|  | myocardial infarction ^57^ | retrovirus | rat BM-MSC | rat | intramyocardial injection,  2×10^6^ cells,  once | ↑ heart structure recovery (↓infarct size)  ↑ angiogenesis (↑vessel densities) |
|  | myocardial infarction ^67^ | pIRES2 plasmid | rat endothelial progenitor cells | rat | intravenous injection,  2 × 10^8^ cells,  once | ↑ engraftment efficiency  ↑ angiogenesis (↑re-reendothelialization) |
|  | myocardial infarction ^68^ | gene-delivery nano system | rat BM-MSC | rat | intramyocardial injection,  2×10^6^ cells,  once | ↓ fibrosis (↓scar area,)  ↑ heart function recovery  ↑ angiogenesis (↑capillary counts, ↑arteriole counts) ↓ cardiomyocytes apoptosis |
|  | myocardial ischemia ^26^ | adenovirus | rat BM-MSC | rat | intramyocardial injection,  5×10^6^ cells,  once | ↑ heart structure recovery (↓infarct size)  ↓ fibrosis (↓collagen content) ↑ angiogenesis (↑capillaries count) ↑ cardiomyocytes proliferation  ↓ cardiomyocytes apoptosis (↑Bcl-2) |
|  | myocardial ischemia ^84^ | adenovirus | rat skeletal myoblast | rat | intramyocardial injection,  5×10^6^ cells,  once | ↑ engraftment efficiency ↑ angiogenesis (↑HGF, ↑VEGF)  ↓ cardiomyocytes apoptosis (↑Bcl-2) |
| lung | pulmonary arterial hypertension ^65,74^ | adenovirus | rat BM-MSC | rat | jugular vein injection,  5×10^6^ cells,  once | ↑ heart structure recovery (↓ET-1, ↓RV/LV ratios)  ↓ fibrosis (↓TGF-β1, ↑MMP-9)  ↑ angiogenesis (↑capillary density, ↑VCAM-1) ↑ cardiomyocytes proliferation (↓nuclear counts) |
|  | bronchiolitis obliterans ^55^ | adenovirus | human UC-MSC | mouse | intravenous injection,  1×10^6^ cells,  once | ↑ lung structure recovery (↓obstruction, ↓occlusion)  ↑ re-epithelialization (↓epithelial apoptosis)  ↓ fibrosis (↓TGF-β) ↓ inflammation (↓IFN-γ, ↑IL-4, ↑IL-10, ↓Treg, ↓Th1/Th2) |
|  | interstitial pneumonia ^72^ | pCikh plasmid | rat BM-MSC | rat | intratracheal instillation,  3×10^6^ cells,  once | ↑ lung structure recovery  ↓ fibrosis (↓hydroxyproline, ↓collagen content) |
|  | acute lung injury ^28^ | adenovirus | rat BM-MSC | rat | intravenous injection,  1×10^6^ cells,  once | ↑ lung structure recovery (↓obstruction)  ↓ lung cell apoptosis  ↓ inflammation (↓MPO, ↓TNF-α, ↑IL-10) ↓ oxidation (↓MDA, ↑SOD) |
|  | radiation-induced lung injury ^29^ | adenovirus | human BM-MSC | mouse | intravenous injection,  1×10^6^ cells,  once | ↑ engraftment efficiency  ↑ lung structure recovery (↓hemorrhage, ↓permeability)  ↑ re-epithelialization (↑proliferation ↑, ↓epithelial apoptosis)  ↓ fibrosis (↓septa thickness, ↓collagen deposition, ↓TGF-β)  ↓ inflammation (↓TNF-α, ↓IFN-γ, ↓IL-6, ↓ICAM-1, ↑IL-10, ↑S1PR1 expression |
|  | acute respiratory distress syndrome ^73^ | adenovirus | human DPSC | mouse | intravenous injection,  1×10^6^ cells,  once | ↑ lung structure recovery (↓obstruction)  ↓ fibrosis  ↓ inflammation |
| liver | radiation induced liver damage ^75^ | lentivirus | rat AD-MSC | rat | intravenous injection,  1×10^7^ cells,  once | ↑ engraftment efficiency  ↓ fibrosis (↓collagen deposition, ↓α-SMA)  ↑ hepatocyte proliferation ↓ hepatocyte apoptosis |
|  | liver fibrosis ^53^ | adenovirus | rat BM-MSC | rat | intravenous injection,  1×10^6^ cells,  once | ↑ engraftment efficiency  ↑ liver function recovery  ↑ hepatocyte activities |
|  | liver fibrosis ^76^ | adenovirus | human BM-MSC | rat | splenic injection,  1×10^7^ cells,  once | ↑ engraftment efficiency  ↑ liver structure recovery (↓damage, ↓PDGF-bb)  ↓ fibrosis (↓Ito cells, ↓COL I, ↓TGF-β1, ↑MMP-9/13/14) |
|  | liver fibrosis ^108^ | adenovirus | human BM-MSC | rat | superior mesenteric vein injection,  4×10^6^ cells,  once | ↑ engraftment efficiency  ↑ liver structure recovery (↓damage) |
|  | liver fibrosis ^78^ | lentivirus | human UC-MSC | rat | intraperitoneal injection,  1×10^6^ cells,  once | ↓ fibrosis (↓COL I, ↓COL III, ↓TGF-β1, ↓α-SMA, ↓Smad2/3) |
|  | liver fibrosis ^56^ | pMEX plasmid | human UCB-MSC | rat | intravenous injection,  1×10^7^ cells,  once | ↓ fibrosis (↓LW/BM ratio) |
|  | liver fibrosis ^52^ | pMEX plasmid | human UCB-MSC | rat | intravenous injection,  2×10^6^ cells,  once a week for 4 weeks | ↓ fibrosis (↓LW/BM ratio, ↓collagen fibers) |
|  | liver fibrosis ^77^ | spermine-pullulan plasmid | rat BM-MSC | rat | intravenous injection,  1×10^6^ cells,  once a week for 3 weeks | ↑ engraftment efficiency  ↓ fibrosis |
|  | acute liver failure ^31^ | adenovirus | human UC-MSC | mouse | intravenous injection,  1×10^6^ cells,  once | ↑ liver structure recovery (↓damage) ↓ hepatocyte apoptosis (↑Bcl-2, ↓Bax, ↑P65)  ↓ inflammation (↓TNF-α)  ↓ oxidation (↑GSH, ↑γ-GCS, ↑SOD, ↓MDA) |
|  | liver transplant ^44,47^ | adenovirus | rat BM-MSC | rat | intravenous injection,  5×10^6^ cells,  once | ↑ engraftment efficiency  ↓ fibrosis (↓HSC activities, ↓TGF-β1) ↑ hepatocytes proliferation  ↓ hepatocytes apoptosis |
| renal | acute kidney injury ^30^ | adenovirus | human UC-MSC | rat | left carotid artery injection,  1×10^6^ cells,  once | ↑ engraftment efficiency  ↑ renal structure recovery (↓damage) ↑ renal cell proliferation  ↓ renal cell apoptosis (↓caspase-3)  ↓ inflammation (IL-1β) |
|  | renal fibrosis ^49^ | adenovirus | rat BM-MSC | rat | intravenous injection,   - 1. ml cells,   once | ↓ fibrosis (↓collagen content, ↓FN, ↓α-SMA) |
|  | renal fibrosis ^45^ | adenovirus | rat BM-MSC | rat | intravenous injection,  1×10^6^ cells,  once | ↓ fibrosis (↓α-SMA) |
| ischemia | hindlimb ischemia ^58^ | adenovirus | mouse BM-MSC | mouse | intramuscular injection,  0.3/3/10×10^5^ cells,  once | ↑ angiogenesis (↑vessel count, ↑HGF, ↑FGF-2) |
|  | hindlimb ischemia ^46^ | AAV | mouse AD-MSC | mouse | intramuscular injection,  1×10^6^ cells,  once | ↑ angiogenesis (↑vessel count)  ↑ neurogenesis (↑nerve growth)  ↓ necrosis |
|  | hindlimb ischemia ^51^ | lentivirus | BM-MSC | rat | intramuscular injection,  5×10^6^ cells,  once | ↑ engraftment efficiency  ↑ angiogenesis |
|  | hindlimb ischemia ^59^ | TALEN and TetOn systems | human UCB-MSC | mouse | intramuscular injection,  2×10^7^ cells,  once | ↑ angiogenesis (↑vessel count, ↑endothelial) |
| bone | osteoporosis ^40^ | adenovirus | human DPSC | mouse | intravenous injection,  1×10^6^ cells,  once | ↓ bone loss (↓RANKL)  ↑ bone regeneration (↑OPG) |
|  | osteonecrosis ^48^ | adenovirus | rabbit BM-MSC | rabbit | femoral head infusion,  1×10^6^ cells,  once | ↑ bone regeneration (↓empty lacunae)  ↓ fibrosis (↓Col I in trabeculae)  angiogenesis (↑hematopoietic, ↑vessel count, ↑VEGF, ↑Col I in medullary cavities)  ↑ p-ERK1/2 expression  ↑ p-Akt expression |
| skin | burn wound ^62^ | adenovirus | rat BM-MSC | rat | burn wounds derma injection,  once | ↑ engraftment efficiency ↑ re-epidermalization  ↓ fibrosis (↓collagen content, ↓hydroxyproline) |
|  | psoriasis ^32^ | adenovirus | human DPSC | mouse | intravenous injection,  2×10^6^ cells,  once | ↑ re-epidermalization  ↓ inflammatory infiltration (↓IFN-γ, ↓TNF-α, ↓IL-17A) |
| others | intracerebral hemorrhage ^39^ | lentivirus | human UC-MSC | rat | left lateral ventricle infusion,  6×10^5^ cells,  once | ↑neurogenesis (↓demyelination, ↑remyelination) |
|  | sinonasal wound ^50^ | adenovirus | human UC-MSC | rabbit | ear vein injection,  6×10^6^ cells,  once | ↑ structure recovery (↑cilia)  ↓ fibrosis (↓collagen deposition, ↓TGF-β) |
|  | periodontitis ^38^ | adenovirus | human DPSC | swine | gingival injection,  1×10^7^ cells,  once | ↑ soft tissue regeneration  ↑ bone regeneration |
|  | radiation-induced intestinal injury ^54^ | adenovirus | human UC-MSC | mouse | intravenous injection,  1×10^6^ cells,  once | ↑ engraftment efficiency  ↑ re-epidermalization (↑HO-1, ↑ZO-1)  ↓ inflammation (**↓**IFN-γ**,** ↑IL-10) |
|  | thymus involution model ^37^ | pMEX plasmid | human AD-MSC | rat | intravenous injection,  2×10^6^ cells,  once | ↑ engraftment efficiency  ↑ thymocyte activities (↑number, ↑ progenitors) |
|  | bladder outlet obstruction ^79^ | retrovirus | human BM-MSC | rat | bladder wall local injection,  1×10^6^ cells,  once | ↓ fibrosis (↓collagen area, ↓TGF-β) |
|  | diabetes ^35^ | lentivirus | rat AD-MSC | rat | left corpus cavernosum injection,  2×10^6^ cells,  once | ↑ structure recovery (↑smooth muscle, ↑SMA)  ↑ angiogenesis (↑endothelium)  ↓ apoptosis (↓caspase-3, ↑Bcl-2, ↑Bcl-xl)  ↓ p-Smad2 expression  ↓ p-Akt expression |

HGF: hepatocyte growth factor; AD: adipose tissue; Akt: threonine kinase; ALP: alkaline phosphatase; BM: bone marrow; c-Met: cellular-mesenchymal epithelial transition factor; COL: collagen; Con A: concanavalin; DA: dopamine; DAT: dopamine transporter; DPSC: dental pulp stem cell; ET-1: endothelin-1; FGF: fibroblast growth factor; FN: fibronectin; GSH: antioxidant glutathione; HO-1: heme oxygenase-1; IFN-γ: interferon gama; IL: interleukin; LW/BW: liver weight per body weight ratio; MAP: man arterial pressure; MDA: antioxidant metabolite malondialdehyde; MMP: matrix metallo proteinase; MPO: myeloperoxidase; OPG: osteoprotegerin; PDGF-bb：platelet-derived growth factor-bb; PECAM-1: platelet-endothelial cell adhesion molecule-1; RANKL: receptor activator of nuclear factor kappa B ligand; RV/LV: left ventricle/right ventricle; S1PR1: sphingosine 1-phosphate receptors 1; SOD: superoxide dismutase; TGF-β: transforming growth factor-beta; TH: tyrosine hydroxylase; Th1: T helper 1 cell; Th1: T helper 2 cell; Th17: interleukin 17 (IL-17)-secreting helper T; TNF-α: tumor necrosis factor alpha; Treg: regulatory T cell; UC: umbilical cord; UCB: umbilical cord blood; VCAM: vascular cell adhesion protein; VEGF: vascular endothelial growth factor; ZO-1: zonula occludens 1; α-SMA: alpha-smooth muscle actin; γ-GCS: gama glutamylcysteine synthetase; VS.: versus; =: equal; ↑: improved; ↓: reduced; \: not mentioned.

**REFERENCE:**

107. Guo YH, He JG, Wu JL, et al. Hepatocyte growth factor and granulocyte colony-stimulating factor form a combined neovasculogenic therapy for ischemic cardiomyopathy [Article]. Cytotherapy. 2008;10(8):857-867. <https://doi.org10.1080/14653240802419278>.

108. Lai L, Chen J, Wei X, et al. Transplantation of MSCs Overexpressing HGF into a Rat Model of Liver Fibrosis. Molecular imaging and biology. Feb 2016;18(1):43-51. <https://doi.org10.1007/s11307-015-0869-x>.
